# Expanding the Porous Alumina Engineering Toolbox for Nanooptics: Multiscale Morphological Tuning via Square-Wave Pulse Anodizing

**DOI:** 10.1021/acsomega.6c02015

**Published:** 2026-04-14

**Authors:** Mikhail Pashchanka

**Affiliations:** Department of Chemical Engineering, 42732 Ariel University, Ariel 40700, Israel

## Abstract

Individual control of nanopore diameter, spacing, and hexagonal ordering in porous anodic alumina (PAA) is essential for designing advanced photonic materials and metamaterials, plasmonic surfaces, and SERS substrates. This paper presents a comprehensive investigation of square-wave pulsed direct current (PDC) anodizing for selective morphological optimization across multiple length scales. Conventional potentiostatic (DC) methods inherently couple pore diameter and hexagonal ordering (both dictated by the best self-ordering regimes occurring at specific voltages). In contrast, PDC anodizing decouples these parameters: the DC-to-PDC transition reduces pore size by up to 60%, while pulse frequency governs well-ordered pore array dimensions. Importantly, the PDC approach maintains consistent nonstoichiometric PAA composition, ensuring that visible property changes (e.g., transparency differences) result solely from structural modifications rather than compositional variations. The demonstrated versatility across different electrolyte types and no requirement for complicated aluminum pretreatment make pulse anodizing a promising tool for optimizing PAA-based optical nanostructures.

## Graphical abstract

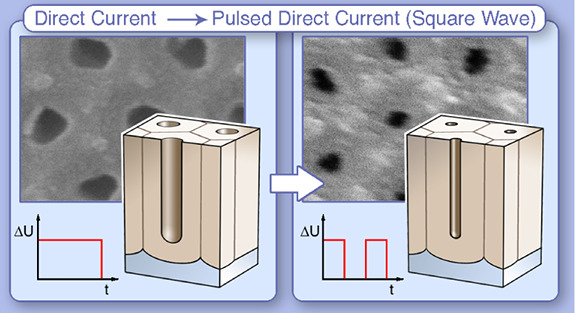

## Introduction

The growing number of applications of porous anodic alumina (PAA) drives advancements in methods for its morphological tuning. One major area of application is nanooptics, which includes subfields like photonics, surface plasmon resonance (SPR), and surface-enhanced Raman scattering (SERS). Nanooptical studies resulted in the development of such PAA-based devices as resonators, interferometers, and label-free biosensors.^1–3^

The pores in anodic alumina can be visualized as cylindrical holes in a dielectric medium forming an inverse two-dimensional (2D) photonic lattice (for 3D lattices with nonconventional pore geometries, see refs 5–7 ).^4–7^ Depending on the degree of long-range order, PAA can exhibit properties of either photonic crystals or disordered 2D photonic systems – the emerging class of materials with significant potential for both fundamental research and future applications.^8–10^ For photonic effects to emerge, the size and spacing of light-scattering elements are essential.^11^ When these parameters are much smaller than the wavelength of the incident light λ, the material behaves like an optically homogeneous medium – e.g., a metamaterial with a tunable refractive index *n*.^12^ Since pore sizes (*d*_p_) and interpore distances (*D*_int_) in self-ordered PAA are adjustable over a wide range (from tens of nm to nearly 1 μm), it is often called a “photonic metamaterial”.^13–16^ While this umbrella term may not be entirely precise from a physics perspective, it highlights PAA’s outstanding versatility for diverse applications. It is worth noting that *n* significantly depends not only on PAA’s morphology but also on its nonstoichiometric composition and impurities, which are determined by formation and post-treatment conditions.^17–19^ Thus, preserving consistent chemical composition while tuning PAA’s geometry is essential for comparing and predicting new architectures with controlled optical properties.

The degree of orderliness is also critical for applications leveraging the unique surface topography of PAA layers or their honeycombed metallic substrates exposed after selective alumina dissolution. Such light-scattering surfaces can be nanostructured solely by anodizing – a reproducible, scalable, and cost-effective technology. Their applications range from structural coloration to SPR and SERS sensing.^20,21^ For example, Kikuchi et al. demonstrated that anodized Al with highly ordered nanotextures exhibits bright structural colors, whereas disordered textures with comparable sizes of light-scattering elements only yield dull grayscale hues.^20^ This observation highlights the potential benefits of exploring controlled regularity and randomness, which could unlock new gradients of visually appealing shades. The size of well-ordered domains also strongly correlates with SERS efficiency, possibly due to constructive interference associated with the hexagonal arrangement of pores.^22^ However, the relationship between individual pore and cell diameters and SERS enhancement is more complex. Unlike SPR, where a change in surface features’ dimensions leads to a systematic signal shift, SERS shows a maximum gain at their specific (efficient) sizes and associated spacing between electromagnetic hot-spots.^23^ For instance, Krohne-Nielsen et al. found that nanopatterned SERS substrates with *D*_int_ = 270 nm exhibited enhancement higher than expected compared to those with periodicities of 100 and 450 nm.^24^ Later, Chen et al. made a similar observation and identified 350 nm pores as optimal for sensing inflammatory biomarkers, while pores of smaller and larger sizes had lower SERS performance.^25^ Thus, multiple studies indicate that a flexible and universal method for selectively modifying *d*_p_, *D*_int_, and the degree of hexagonal pore arrangement is highly desirable across a wide range of cutting-edge research fields.

A recent study showed that pulsed direct current (PDC) anodizing could be a proper method for independent tuning of the aforementioned parameters.^26^ The transition from a direct current (DC) regime to rectangular voltage pulses of equal magnitude reduced the *d*_p_/*D*_int_ ratio by a factor of 1.6–1.9, which reflects changes in PAA porosity and affects related optical properties like the effective refractive index *n*_eff_.^27^ Moreover, varying the pulse frequency *f* allows one to regulate the degree of randomness in the pore layout, thus facilitating a systematic investigation of the related photonic effects (see Figure 1).^10^ The PDC method demonstrates significant advantages over artificial prepatterning techniques (such as imprinting and e-beam lithography) that are fundamentally limited by natural pore rearrangement during extended anodization.^28,29^ Pulsed oxidation enables stable PAA growth with consistent *d*_p_ and *D*_int_ values, allowing fabrication of tailored cylindrical nanochannels with very high aspect ratios.

**Figure 1:**
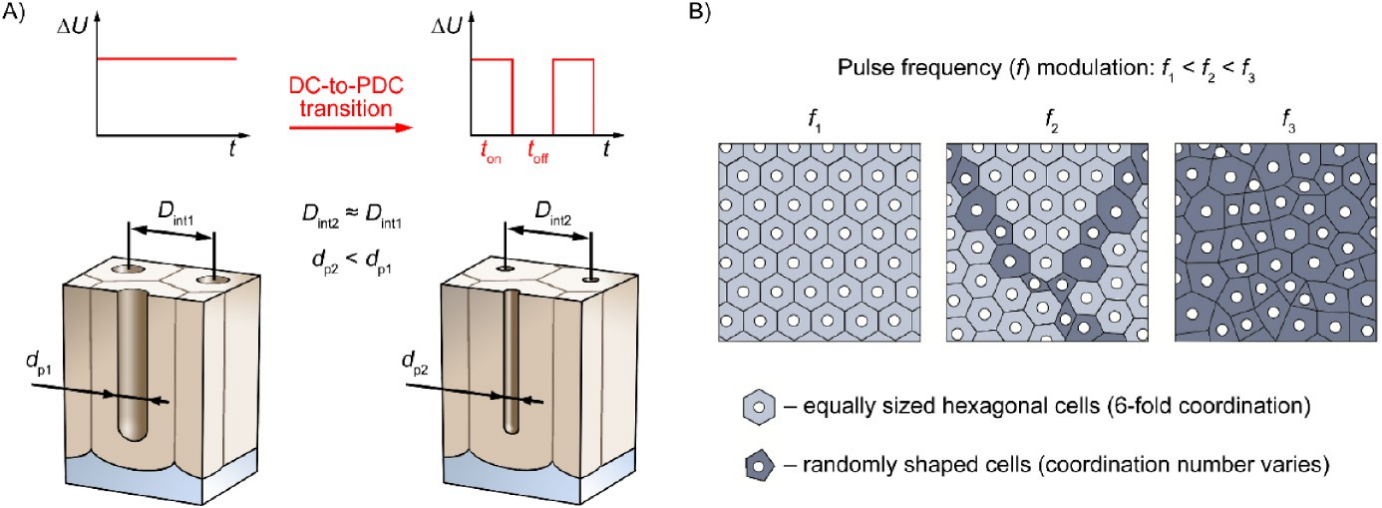
Schematic illustration of PAA morphology control via PDC anodizing with a rectangular waveform (*t*_on_ = *t*_off_). (A) Switching from DC to PDC at the same maximum voltage (Δ*U*) produces straight nanochannels with *d*_p_ reduced approximately by half, while *D*_int_ varies moderately (within 15 ± 10%). (B) Increasing *f* introduces morphological disorder while keeping *d*_p_ and *D*_int_ invariant (although *D*_int_ exhibits a larger spread in values in disordered areas due to pore arrangement irregularity), allowing largely decoupled control of coherent nanopore domain size in materials with fixed porosity.

This study investigates whether rectangular-pulse anodizing can open new avenues for preparing practically relevant and tunable light-scattering materials. Although it focuses on morphological control and quantifiable compositional differences in free-standing transparent and translucent PAA laminas (rather than instrumental analysis of functional properties), the findings have broader implications. For example, they can help create new μm-thick dielectric spacers with adjustable *n*_eff_ for SPR sensors in Otto configuration, or tunable semiordered functional textures on aluminum – either for direct optical applications or for transfer to other moldable materials via nanoimprinting techniques.^20,30^

The paper addresses several key questions related to long-term PDC oxidation:

- Do the desired pore arrangements persist during prolonged anodizing? Previous DC experiments showed that pore ordering improves during the first 20 h, after which it begins to deteriorate.^22,31^ While finding such a universal relationship for PDC regimes requires a dedicated study, it is still valuable to preliminarily assess whether the PDC method is compatible with the time-frames needed to achieve PAA thickness sufficient for mechanical stability.
- To what extent does prolonged contact with reactive, alumina-dissolving electrolytes in situ affect PDC-modulated pore sizes associated with the refractive properties (*n*_eff_)? ^18,27^

- Do PAA samples produced at different frequencies *f* demonstrate comparable nonstoichiometric compositions? Although the dependence of PAA composition on applied voltage parameters is known, previous studies have not primarily focused on the characteristics of PDC-derived films, with only brief mentions in some works.^32–34^ Meanwhile, compositional variations significantly impact fundamental optical properties like absorption coefficient or color tinge (e.g., oxalate anions typically give PAA a yellowish hue).

This experimental work focuses on sub-1-Hz pulse frequencies to test the earlier hypothesis that they better combine the advantages of both the self-ordering and low-voltage regimes (hexagonal arrangement and *d*_p_ decrease).^26^ Additionally, it utilizes 0.3 M H_2_SO_4_ electrolyte solution instead of the previously tested 10 wt % H_2_SO_4_,^26^ yielding different pore and cell dimensions. This confirms the method’s universality and predictable outcomes across electrolyte types, concentrations, and applied voltages.

## Experimental Section

PAA samples were prepared in a stirred and thermostatically controlled (0.5 ± 0.2 °C) electrolytic bath, using a Toellner TOE 8951-130 power supply with an arbitrary function generator and a Julabo F12-MA thermostat. Flat rolled aluminum sheets (99.93 wt %, 1 mm thick) from AMAG Rolling GmbH (Austria) were ultrasonically cleaned in acetone for 10 min, and then used “as is” for equal-sized (several cm^2^) parallel-plate electrodes without prior electropolishing. Two series of experiments were conducted in 0.3 M oxalic acid (H_2_C_2_O_4_) and 0.3 M sulfuric acid (H_2_SO_4_) electrolyte solutions under PDC conditions at frequencies of 1, 0.5, 0.1, 0.05, and 0.01 Hz. The voltage amplitude was alternated between 0 and 40 V for 0.3 M H_2_C_2_O_4_ and 0–27 V for 0.3 M H_2_SO_4_, with equal durations of pulse and relaxation (a square waveform; *t*_on_ = *t*_off_, see Figure 1). These maximum output voltages (Δ*U*) correspond to the optimal self-ordering voltages reported for long-term DC anodizing in each electrolyte.^35,36^ Solutions were prepared using oxalic acid dihydrate (>99 wt %, Thermo Scientific Chemicals) or sulfuric acid (>99 wt %, VWR) dissolved in distilled water (σ ≈ 2 μS cm^–1^). All samples were fabricated using the two-step anodizing process developed by Masuda et al.^35^ The first oxidation at selected frequency was performed for 4 h to flatten aluminum native surface defects and generate a patterned texture with frequency-dependent ordering. The resulting PAA layer was then removed in an etching solution (0.16 M K_2_Cr_2_O_7_ and 1.5 M H_3_PO_4_), after which the main anodizing was conducted at the same frequency for 3 h (except for 72-h fabrication of mechanically stable free-standing films at *f* = 0.1 Hz). For comparison, Al plates were also anodized under conventional DC conditions at the same voltages (40 and 27 V for respective electrolytes) without waveform generation. The first DC anodizing step lasted 2 h to match the total electric charge transferred in the DC and PDC regimes, in accordance with Faraday’s law. The main DC oxidation continued for 20 h in both electrolytes to produce PAA films of sufficient thickness for more accurate growth rate comparison. PAA films were detached from substrates using the polarity reversal method; for PDC-derived films, the process was switched to DC mode prior to separation.^37,38^ Microscopic examination and energy-dispersive X-ray analysis (EDX) were performed on a Philips XL-30FEG field emission scanning electron microscope – SEM (20 kV accelerating voltage, 5 mm working distance, spot size 3). Samples were mounted on conductive carbon-rich polymer film and coated with 4.5 nm Pt/Pd alloy by magnetron sputtering. For statistical analysis of average pore and cell dimensions (*d*_p_ and *D*_int_), ten random measurements were taken from SEM images, with confidence intervals calculated at a significance level α = 0.05. All calculated values were rounded to whole nanometers to match the resolution limits of the microscope. The color-coding of ordered and disordered pore arrays on low-magnification SEM micrographs was based on careful analysis of individual pore coordination numbers and the presence of defects, and was performed manually using raster image editing software. Individual and total surface areas of well-ordered domains were determined using the SketchAndCalc irregular area calculator (see the Supporting Information file for details). Thermogravimetric analysis (TGA), derivative thermogravimetry (DTG), and differential thermal analysis (DTA) were carried out using a Netzsch STA 449 C Jupiter thermo-microbalance, with a heating rate of 10 °C min^–1^ from ambient temperature to 1300 °C in synthetic air (flow rate 75 mL min^–1^).

## Results and Discussion

### SEM Characterization and Quantification of Morphological Parameters for PDC-Derived PAA

For a more detailed understanding of the effect of sub-1-Hz pulse frequencies on PAA morphology, microscopic examination of the samples obtained from two different electrolyte solutions at progressively decreased *f* values (1–0.01 Hz) was carried out. The SEM results from the series of PDC experiments conducted in 0.3 M oxalic acid at Δ*U* = 40 V are summarized in Figure 2 High-magnification micrographs of the porous surface provide a closer look at individual PAA cells and enable statistical estimation of the average *d*_p_ and *D*_int_ values (quantified in Figure 4A and B). Overall view images in the central column of Figure 2 elucidate the self-ordering behavior. Fractured cross-section images in the last column confirm the absence of significant morphological distortions in the PDC-generated PAA channels when compared to those formed under the DC regime (e.g., variation in *d*_p_ along pore axes or increased pore branching). It should again be noted that straight, parallel cylinders represent an idealized model of nanopores in self-ordered anodic alumina. As recently demonstrated by Ono and Asoh, smaller radially distributed pores and protrusions can form within the main cylindrical channels even under best self-ordering DC conditions in 0.3 M H_2_C_2_O_4_ at Δ*U* = 40 V.^39^ Thus, pore growth is characterized here only on the mesoscopic scale, without addressing finer structural details. In the middle column of Figure 2, color-coded areas represent well-ordered nanopore domains (cyan) and disordered regions (orange). Pore classification is based on analysis of coordination numbers: pores with six nearest neighbors were assigned to hexagonally ordered arrays (even in cases of moderate lattice distortion), while those surrounded by 5, 7, or 8 neighbors were categorized as disordered. Defects like missing pores or double pores within a single cell were also classified as elements of disorder. It should be noted that this division of domains is inevitably somewhat arbitrary. For instance, pores located at the boundaries between ordered and disordered regions may be assigned to either category. Additionally, isolated point defects within highly ordered domains complicate their precise surface area calculation. Nevertheless, this method has demonstrated effectiveness in preceding studies, correlating with established self-ordering mechanisms and orderliness-dependent measurable optical properties, such as SERS efficiency.^22,40^ It was previously established that anodization at higher frequencies (10–10^6^ Hz) results in highly random pore layouts, whereas signs of incipient long-range ordering appear at lower *f* values.^26^ Therefore, the present article focuses on investigating the potential of applying pulse frequencies below 1 Hz for fine-tuning the proportions of well-ordered and disordered areas, and on providing an initial assessment of the viability of this approach. As shown in Figure 2, the application of all examined pulse frequencies results in the formation of semiordered porous layers. When transitioning from 1 to 0.5 Hz, the total coverage of well-ordered domains remains nearly unchanged, with only a modest increase from 27.0% to 28.3%, as quantified from SEM data (in every case, the total analyzed surface area was ≈ 4 × 10^6^ nm^2^ – see Figures S2–S11 in the Supporting Information file for computational details). Upon subsequent decrease in the frequency from 0.5 to 0.1 Hz, the fraction of ordered domains increases substantially, reaching 40.4% (Figure S4). Notably, this increase is primarily due to a higher number of small, short-range ordered fragments, which may serve as nucleation sites for the subsequent growth of larger domains. A further decrease down to *f* = 0.05 Hz results in a reduction in the number of domains, accompanied by a shift toward a larger average domain size due to the diminished presence of the smallest hexagonal fragments. This consolidation process (i.e., the merging of smaller hexagonal arrays into larger ones) is, however, associated with a decrease in the overall ordered area down to 34.0% (Figure S5). At the lowest frequency tested (0.01 Hz), the PAA samples exhibit further enlargement and coalescence of well-ordered domains, with the total ordered surface area increasing to 49.2% (Figure S6). Although a robust statistical analysis is not yet available at this stage, this increase should, nonetheless, be regarded as significant. While a more thorough investigation with a larger number of compared samples and varied parameters (such as preanodizing durations) is necessary to obtain an accurate statistical picture of the self-ordering behavior in PDC-derived PAA, these preliminary results already allow for some predictions regarding the evolution of self-organization. The process appears to be nonlinear, characterized by alternating phases of domain nucleation and growth, accompanied by parallel fluctuations in the fraction of ordered areas.

**Figure 2:**
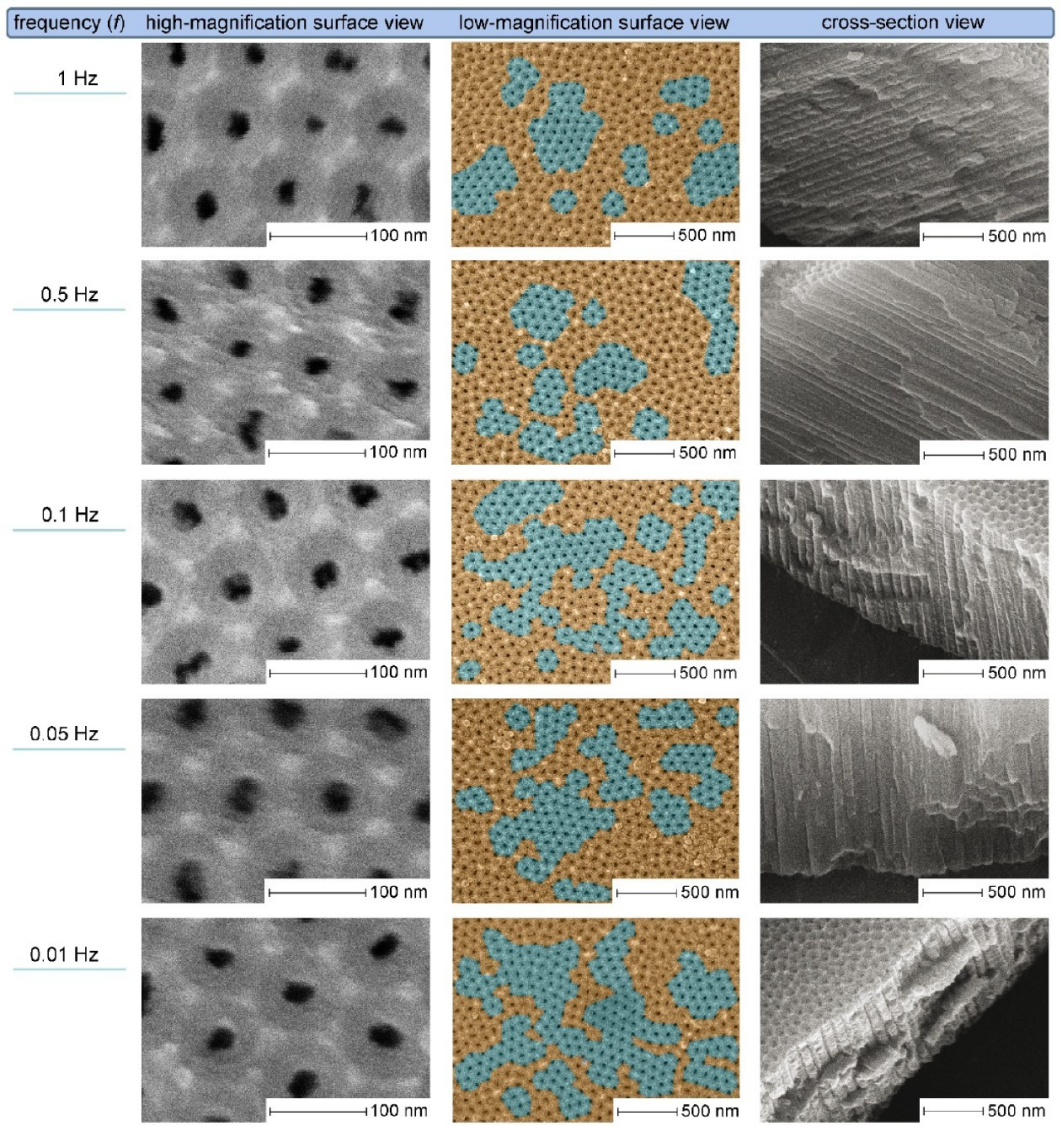
SEM micrographs of PAA layers fabricated in 0.3 M H_2_C_2_O_4_ using PDC anodizing with square waveform, 40 V peak voltage, and variable pulse frequencies from 1 to 0.01 Hz. All images were acquired at consistent magnifications of 80,000× (overall porous side and fractured cross-sectional views) and 650,000× (nanopore close-ups). Hexagonally ordered pore domains are colored in cyan, while disordered areas are highlighted in orange. Quantitative results of domain area analysis, as well as a comprehensive description of the methodology are available in the Supporting Information.

Figure 3 presents the results of an analogous microscopic study of PAA layers fabricated in a 0.3 M sulfuric acid electrolyte. As noted in the introduction, investigation of mild PDC anodizing with this previously untested H_2_SO_4_ concentration confirms the universality of the observed self-ordering patterns. All samples in this series exhibit a higher degree of order compared to “oxalic” PAA layers, likely due to the higher current density from the stronger electrolyte. Improved charge transport properties in H_2_SO_4_ solution accelerate electrochemical reactions, thereby promoting faster evolution of ordered surface patterns. Similar to “oxalic” PAA, cross-sectional images of samples from 0.3 M H_2_SO_4_ reveal equidistant straight nanochannels with no apparent morphological defects. The sample anodized at *f* = 1 Hz shows a relatively high ordered domain coverage (41.5%; see Figure S7 in the Supporting Information for details). Reducing the frequency to 0.5 Hz substantially increases both the sizes of individual domains and the overall orderliness (59.6% surface coverage, Figure S8). At *f* = 0.1 Hz, a minimal decrease in the degree of order (down to 58.3%, Figure S9) is observed, accompanied by the onset of large domains disintegration. This trend continues at lower frequencies, where nucleation of new small domains occurs in parallel with a systematic reduction in ordered surface area: 51.7% at 0.05 Hz and 44.4% at 0.01 Hz, respectively (Figures S10–S11). There are sufficient grounds to assume that further nonlinear effects may occur in 0.3 M H_2_SO_4_ at frequencies beyond the studied range (e.g., below 0.01 Hz). For instance, this could involve a reversal in trends toward renewed domain consolidation and improved orderliness, similar to that previously observed for “oxalic” PAA between 0.05 and 0.01 Hz.

**Figure 3:**
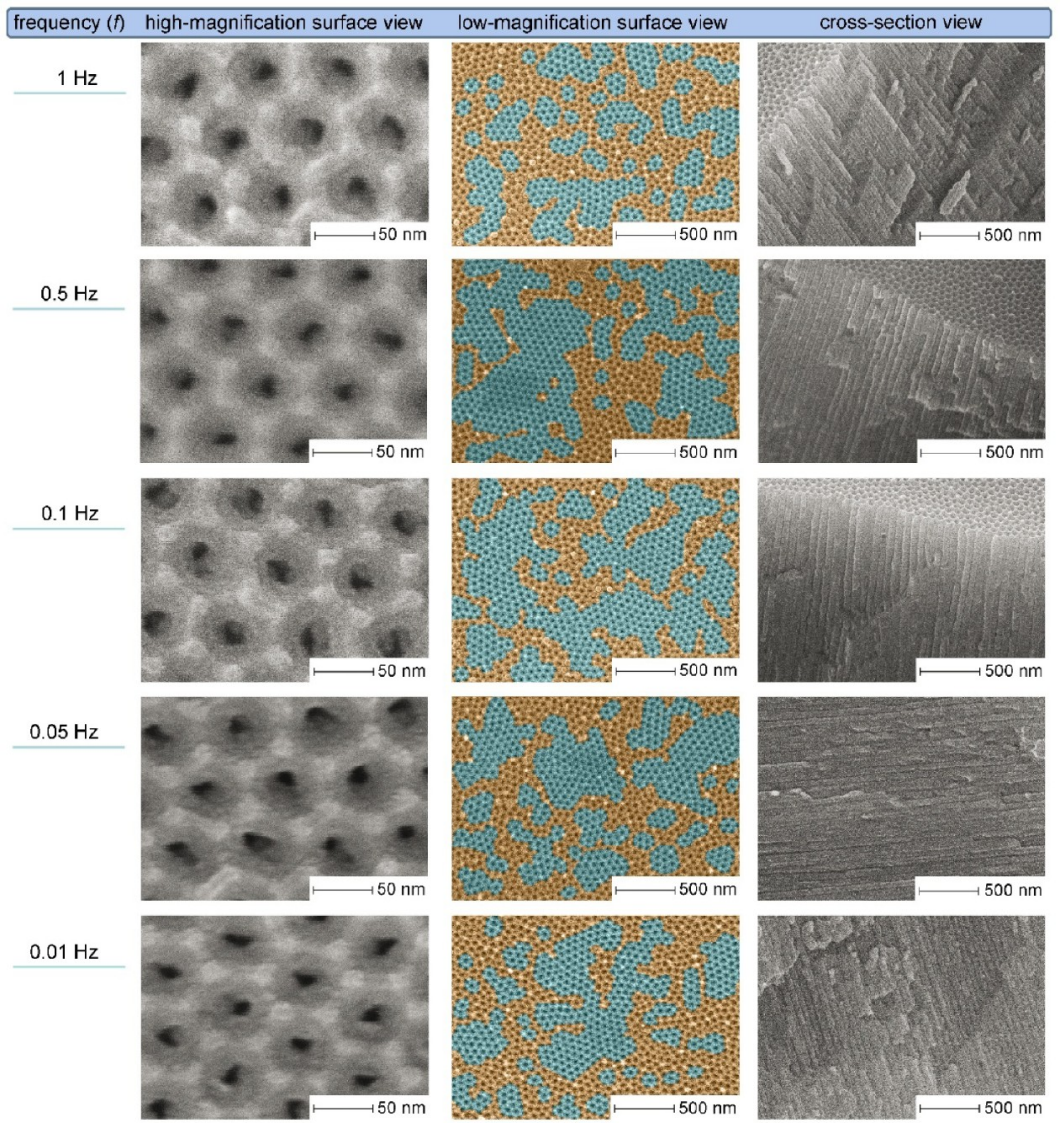
SEM images of PAA laminas produced by PDC anodizing in 0.3 M H_2_SO_4_ electrolyte at a maximum output voltage of 27 V, with pulse frequencies ranging from 1 to 0.01 Hz. Close-up images were acquired at 800,000× magnification, while overall views of the porous surface and cross sections were taken at 80,000×. The color-coding for ordered (cyan) and disordered (orange) regions follows the scheme from Figure 2. Quantitative data from the domain surface area analysis are provided in Figures S7–S11 in the Supporting Information file.

Here it is necessary to make a short digression and emphasize that there is currently a broad variety of automated approaches to assessing the degree of ordering in PAA in the literature, including fast Fourier transform (FFT), Voronoi tessellation, analysis of deviations of triangular pore constellations from equilateral geometry, or a recently proposed mean angle displacement (MAD) method.^41–44^ Such methods continue to evolve as their parameters and algorithms are refined with the aim of achieving greater mathematical objectivity. However, no method is currently considered a “gold standard” in hexagonal order assessment, and methodological choices based on specific analytical tasks remain a researcher’s prerogative. Given the current rapid advancement in digital technologies, it can be anticipated that hexagonal ordering analysis will be further enhanced through artificial intelligence (AI)-based approaches, which, despite their mathematical complexity, may substantially streamline such routine analytical tasks and increase research productivity.

The results of statistical analysis of pore diameters *d*_p_ and interpore spacing *D*_int_, measured from high-resolution SEM images, as well as the calculated *d*_p_/*D*_int_ ratios and surface porosities are presented graphically in Figure 4. Empirical interpolations of the *d*_p_ values suggest a universal dependence between pore diameters and the applied pulse frequencies (see Figure 4A). However, the deviations of *d*_p_ toward smaller values at *f* = 0.05 Hz, observed in both electrolytes, may indicate that the exact relationships may not fully conform to the smooth curve fits shown in the plots (e.g., due to a complex, nonlinear interplay between frequency-dependent ion transport, relaxation processes, and kinetics of electrode reactions that largely define the final pore dimensions). For enhanced visualization of these deviations in the lower frequency range, the data from Figure 4 are also presented with logarithmic *f* scaling in Figure S1 in the Supporting Information. For PAA samples obtained from oxalic acid, the largest mean pore diameter of 24 ± 2 nm was observed at the lowest applied pulse frequency *f* = 0.01 Hz, which corresponds to equal anodizing and relaxation periods of 100 s. The smallest mean pore sizes of 17 ± 2 and 17 ± 4 nm were measured at frequencies of 0.5 and 1 Hz, respectively. It should be noted that in cases where pore openings were asymmetrical, the maximum internal pore diameter (i.e., the largest distance between opposite pore walls) was measured and used for statistical calculations. DC-generated pores in 0.3 M H_2_C_2_O_4_ at Δ*U* = 40 V typically measure about 31 nm in diameter (without widening post-treatment).^31^ Thus, modulation of *f* in the PDC method reduces *d*_p_ by a factor of approximately 1.3–1.8 compared with conventional DC anodizing under equivalent conditions (electrolyte composition, temperature, and Δ*U*).

**Figure 4:**
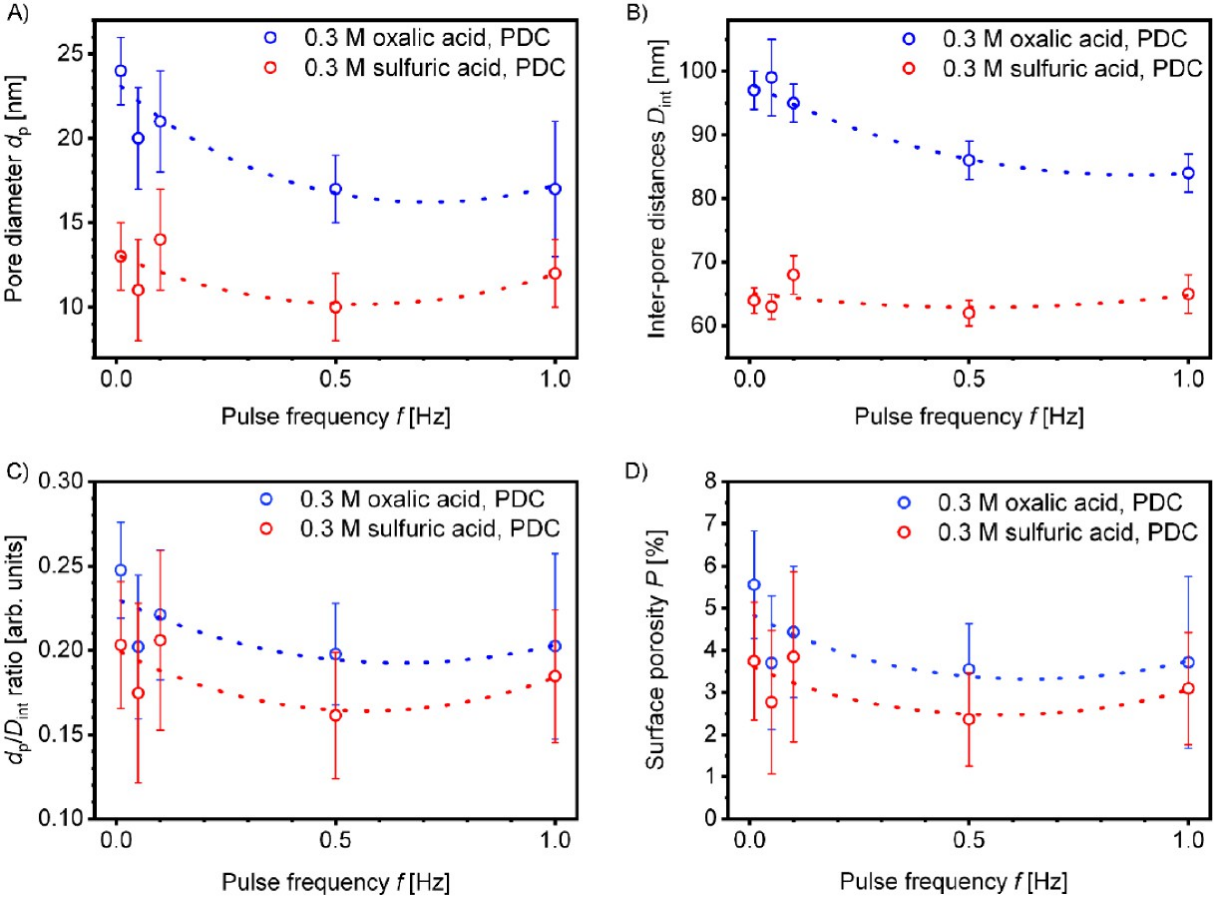
Dependencies of pore diameters *d*_p_ (A), interpore distances *D*_int_ (B), *d*_p_/*D*_int_ ratios (C), and surface porosities (D) on voltage pulse frequencies *f* for PAA layers formed in 0.3 M H_2_C_2_O_4_ and 0.3 M H_2_SO_4_ electrolyte solutions under PDC anodizing conditions at maximum output voltages of 40 and 27 V, respectively. Interpolation curves represent best fits accounting for statistical evaluation errors. See Figure S1 in the Supporting Information for data presented on a logarithmic *f* scale for improved visualization in the low-frequency range.

For 0.3 M H_2_SO_4_, the largest pore diameters of 13 ± 2 and 14 ± 3 nm were observed at *f* = 0.01 and 0.1 Hz, respectively, while the smallest *d*_p_ of 10 ± 2 nm corresponded to *f* = 0.5 Hz. The original work by Masuda et al. (which primarily focuses on optimizing DC conditions in 0.3 M H_2_SO_4_ for achieving ideal long-range hexagonal ordering) reports no *d*_p_ value for Δ*U* = 27 V.^36^ However, *d*_p_ = 24 nm has been reported by Nielsch et al. for PAA films fabricated at a slightly lower constant voltage of 25 V.^31^ It is reasonable to expect *d*_p_ values just above 24 nm for potentiostatic anodizing at 27 V. Therefore, the controllable ratio between pore sizes obtained under PDC and DC conditions is somewhat greater than 1.7–2.4. Remarkably, the DC-to-PDC transition results in a more pronounced reduction of *d*_p_ in H_2_SO_4_ electrolyte compared to H_2_C_2_O_4_. This could be explained by a stronger suppression of local alumina dissolution in sulfuric acid due to relaxation periods and diffusion-driven pH increase at the pore bottoms.

The interpore distances *D*_int_ for PAA formed in different electrolytes under DC conditions are well-characterized in the literature. For 0.3 M oxalic acid at Δ*U* = 40 V, the reported *D*_int_ values are 99 ± 8 nm (Masuda and Fukuda), 100 nm (Asoh et al.), and 105 nm (Nielsch et al.).^31,35,45^ For 0.3 M sulfuric acid, *D*_int_ values of 63 and 66.3 nm have been reported for potentiostatic anodizing at 25 V.^31,45^ However, using the well-known empirical proportionality factor between *D*_int_ and the applied voltage (≈2.5), it can be estimated that Δ*U* = 27 V yields a hexagonal lattice with a periodicity of 67.5 nm. Under PDC conditions, the interpore distances vary between 99 ± 6 and 84 ± 3 nm for oxalic acid (at 0.05 and 1 Hz, respectively), and between 68 ± 3 and 62 ± 2 nm for sulfuric acid (0.1 and 0.5 Hz; see Figure 4B). Thus, the maximum cell contraction is only by a factor of approximately 1.18–1.25 for oxalic acid and less than 1.09 for sulfuric acid. In other words, the changes in *D*_int_ for “oxalic” PAA appear to be relatively minor compared to the changes observed in *d*_p_, while the changes in *D*_int_ for “sulfuric” PAA can be regarded as negligible.

As displayed in Figure 4C, the *d*_p_/*D*_int_ ratios vary within a narrow range, from 0.25 ± 0.03 to 0.20 ± 0.03 for PDC-derived “oxalic” PAA, and from 0.21 ± 0.05 to 0.16 ± 0.03 for “sulfuric” PAA. Taking into account the ratios of 0.30 and 0.36, respectively, for DC-derived “oxalic” and “sulfuric” laminas (based on measurements reported by Nielsch et al.), it is evident that DC-to-PDC transition results in a considerable decrease in the *d*_p_/*D*_int_ values for both electrolytes.^31^ This reduction enables additional control over porosity-related properties, such as the effective refractive index (*n*_eff_).

Image-based porosity estimation using *d*_p_ and *D*_int_ values is a common method for analyzing PAA morphology (see Figure 4D). For example, results of such calculations underpin the hypothesis that optimal self-ordering regimes yield PAA layers with porosities around 10%, while disordered aluminas exhibit significantly higher or lower values.^31^ However, there are certain exceptions to this “10% porosity rule”, often explained by variations in alumina dissolution rates under different self-ordering conditions. For example, DC anodizing in 0.3 M H_2_C_2_O_4_ at 40 V and temperatures of 1, 15, and 20 °C yields *d*_p_ = 31, 63, and 50 nm, and *D*_int_ = 105, 100, and 92 nm, respectively.^46^ Thus, *d*_p_ can be doubled at a given self-ordering voltage, while *D*_int_ changes by only about 12%, enabling porosity variation. Stępniowski et al. and Christoulaki et al. have shown that *d*_p_ is controlled by the temperature and duration of anodizing, while *D*_int_ depends primarily on Δ*U*.^47,48^ The conclusions of these two studies reinforce previous findings and suggest that porosities significantly different from 10% are not necessarily a sign of limited potential for geometry optimization in a self-organized PAA structure.

The confidence intervals for porosities demonstrated in Figure 4D are derived from *d*_p_ and *D*_int_ estimates (Figure 4A and B) by calculating the percentage of pore area relative to cell area. Pore areas were calculated as π*r*_p_^2^, where *r*_p_ = *d*_p_/2 is the mean pore radius. Cell areas were obtained as $2\sqrt{3}a^{2}$ , where *a* = *D*_int_/2 is the apothem of a hexagonal cell. A slightly larger dispersion in irregular cell sizes is taken into consideration: their average dimensions are presumed to be close to those of regular cells, although the confidence intervals may be wider. As Figure 4D indicates, the porosities of both “sulfuric” and “oxalic” PDC-derived PAAs are similar in value and exhibit comparable systematic changes with varying applied frequency *f*. The highest porosity value for “oxalic” PAA reaches 5.6 ± 1.3% at *f* = 0.01 Hz, while the lowest is 3.5 ± 1.1% at *f* = 0.5 Hz. The corresponding values for PAA formed in 0.3 M H_2_SO_4_ are 3.7 ± 1.4% and 2.4 ± 1.1% at *f* = 0.01 and 0.5 Hz, respectively. For the H_2_SO_4_-based system, two points are displaced from the interpolated curve: 2.8 ± 1.7% at *f* = 0.05 Hz and 3.8 ± 2.0% at *f* = 0.1 Hz, but their error bars remain consistent with the overall trend (see Figure S1D in the Supporting Information for detailed visualization on a logarithmic *f* scale). As evident from comparison with the 8–12% porosities of the corresponding DC-derived layers, switching to PDC anodizing leads to a pronounced reduction in porosity – by a factor up to 5 in the 0.3 M H_2_SO_4_ electrolyte.^31^ A similarly low porosity of 3.5% was reported by Moyen et al. using a galvanostatic pretreatment of Al for pore nucleation followed by a potentiostatic PAA growth.^49^ However, the main oxidation step duration in their study was limited to 150 s, which may reflect pore dimension alterations during prolonged DC anodizing. In contrast, the new PDC approach demonstrates potential for fabricating low-porosity PAA with high-aspect-ratio nanochannels (the duration of the experiments summarized in Figure 4 was 72 times longer, or 36 times if only the current-on periods are considered).

### Long-Term PDC Anodizing: Free-Standing Film Fabrication and Growth Kinetics Assessment

Considering the possible need for prolonged anodizing for the enhancement of the PAA layers’ mechanical stability, and the simultaneous risk of *d*_p_ increase due to extended exposure to aggressive solutions, a systematic study of films obtained via 72 h PDC oxidation (*f* = 0.1 Hz in both electrolytes) and their comparison with DC-derived counterparts has been undertaken. The results presented below address three main aspects: the feasibility of unsupported laminas fabrication using the polarity reversal approach, the changes in pore opening sizes due to chemical dissolution, and analysis of the film growth rates under both PDC and DC regimes.

As shown in Figure 5A, PDC-derived laminas with substantial surface area can be effectively detached from Al substrates by reversing the electrode polarity.^37,38^ For optimal hydrogen evolution and improved film separation, the systems were switched to DC mode during this step. Photographs reveal that both types of PAA transition from predominantly transparent to translucent when switching from DC to PDC fabrication (a black, light-absorbing background is used in Figure 5B to minimize reflected light). Tilted-view images (Figure 5C) highlight the glossy appearance of the top surface, indicating that light scattering occurs mainly within the PDC-derived PAA rather than at the air/PAA interface. It is well established that optical transparency is influenced by microscale grains, grain boundaries, morphological defects, porosity, and other light-scattering centers, which are most effective when their dimensions are comparable to visible wavelengths (hundreds of nm). Since the characteristic size of a single mesoscopic PAA cell is much smaller than visible light wavelengths, the observed changes in scattering cannot be attributed solely to nanopore arrangement. It is possible that nanopores within an ordered domain collectively act as a coherent unit, with scattering occurring at domain boundaries. Additionally, slight contrasts in the effective refractive indices *n*_eff_ between highly ordered and disordered regions may further enhance light refraction at their interfaces. This may explain the difference in transparency between DC- and PDC-derived laminas based on their ordered versus semiordered structures (pending optical validation). This difference possibly relates to spatial variations in pore ordering at the micrometer rather than nanometer level.

**Figure 5:**
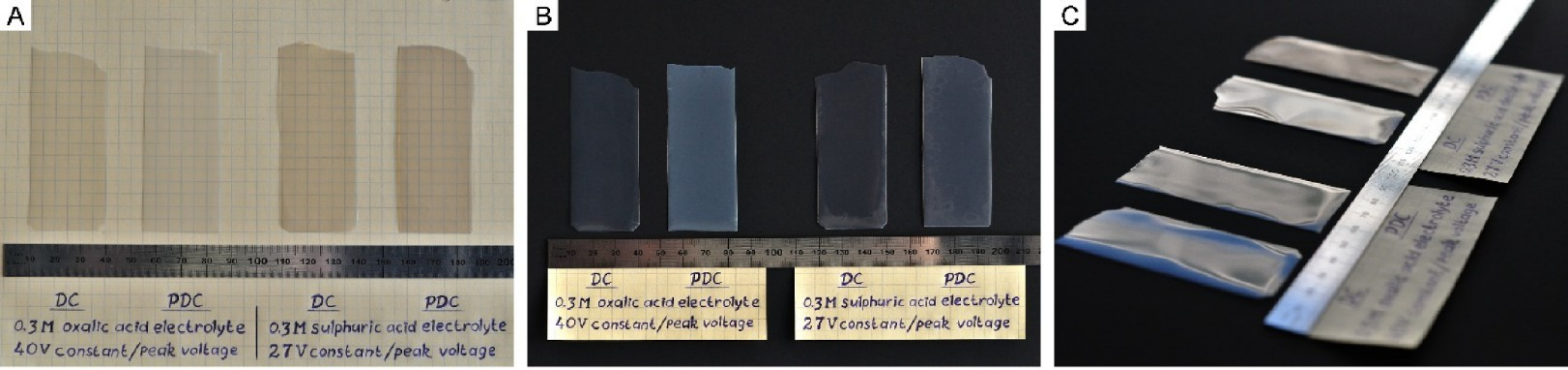
Side-by-side comparison of free-standing PAA films fabricated in 0.3 M H_2_C_2_O_4_ and 0.3 M H_2_SO_4_ solutions under DC and PDC (*f* = 0.1 Hz) conditions. All samples have a surface area of 22–23 cm^2^ (A). Initial visual assessment shows that PAA obtained from both electrolytes transitions from transparent to translucent upon switching from DC to PDC regimes (A, B). Photographs taken on a matte black, light-absorbing surface (B) highlight the enhanced internal light scattering in PDC-derived films, indicative of a higher density of structural imperfections. Tilted-view images (C) demonstrate glossy film surfaces, confirming that the observed optical changes primarily result from increased internal scattering rather than surface roughness.

Although the changes in light-scattering properties demonstrated in Figure 5 still await quantitative characterization via advanced (e.g., angle-resolved) optical methods, it is worthwhile to discuss the practical relevance of the PDC anodizing approach based on the initial qualitative observations. For instance, it is common knowledge that applications of decorative protective coatings depend on the transparency of PAA films that are typically produced in sulfuric acid and preserve the brilliance of an adequately polished surface of aluminum or its customized alloys.^50^ If optical transparency is not a priority, then a less concentrated electrolyte solution and a different set of anodizing conditions (i.e., a lower temperature and a higher current density that needs to be controlled by using a dummy workpiece as a sensor) are usually needed for obtaining dull protective films.^50^ In the realistic situation of small-batch production where customers may prefer glossy or matte (“satin”) decorative surface finishes, the PDC approach can facilitate easy switching between the two decorative effects without the necessity to reformulate electrolyte solutions and refill large-volume baths of automated anodizing lines. Additional mechanical and chemical metal polishing steps to create products with different aesthetic properties would also not be required. Thus, the PDC approach may potentially help small- or medium-sized enterprises optimize their production processes by simplifying existing commercial anodizing protocols, thereby bringing in new economic standards to the sector.

Figure 6 presents a detailed comparison of SEM images of the unsupported PAA samples fabricated under different anodizing conditions. The most striking observation is that pore openings in both “oxalic” and “sulfuric” PDC-generated films (Figure 6F and P) are significantly larger after 72 h of anodizing compared to 3 h (Figures 2 and 3), and exceed those observed in samples after 20 h of DC anodizing (Figure 6A and K). The quantitative estimates of *d*_p_, *D*_int_, and growth rates for all PAA samples displayed in Figure 6 are summarized in Table 1. The pore sizes observed in DC-generated PAA closely match previously reported values (e.g., by Nielsch et al.).^31^ In the prolonged PDC experiments, pore diameters increase by factors of approximately 2.0 for oxalic acid and 2.8 for sulfuric acid electrolytes (relative to values shown for *f* = 0.1 Hz in Figure 4A). These results confirm that prolonged exposure to aggressive electrolyte solutions significantly affects pore dimensions and must be considered when designing and fine-tuning PAA structures. However, the maximum thickness of the layers required for nanooptical applications, such as highly uniform dielectric spacers for SPR sensors in Otto configuration, typically measures only a few micrometers.^30^ Consequently, the extremely long anodizing periods used in these tests will probably rarely be needed in practical applications. High-resolution SEM images in Figure 6C–D,H–I,M–N and R–S show clean bottom sides (barrier layers) of PAA, with virtually no remaining metallic aluminum and clear visibility of nanoscale morphological features. This demonstrates that the polarity reversal technique is highly effective for separating PAA from Al substrates. For significantly thinner or more brittle laminas, alternative methods such as aluminum amalgamation with HgCl_2_ or dissolution in a CuCl_2_ and HCl solution may also be used. As shown in Table 1, the measured *D*_int_ values for PAA layers obtained under DC regimes closely match earlier data published elsewhere.^31^ Under PDC regimes, the *D*_int_ values agree with those presented in Figure 4B for *f* = 0.1 Hz (95 ± 3 nm for oxalic acid and 68 ± 3 nm for sulfuric acid, respectively).

**Table 1:** Morphological Parameters (*d*_p_, *D*_int_) and Growth Rates of Free-Standing PAA Layers Synthesized in 0.3 M Oxalic and Sulfuric Acid Solutions under DC (20 h) and PDC (72 h, *f* = 0.1 Hz) Conditions

PAA type	*d*_p_ (nm)	*D*_int_ (nm)	Growth rate (μm h^–1^)
0.3 M H_2_C_2_O_4_, DC	35 ± 5	105 ± 3	1.985
0.3 M H_2_C_2_O_4_, PDC	41 ± 4	94 ± 5	0.969
0.3 M H_2_SO_4_, DC	23 ± 2	67 ± 2	4.645
0.3 M H_2_SO_4_, PDC	39 ± 2	69 ± 4	2.306

**Figure 6:**
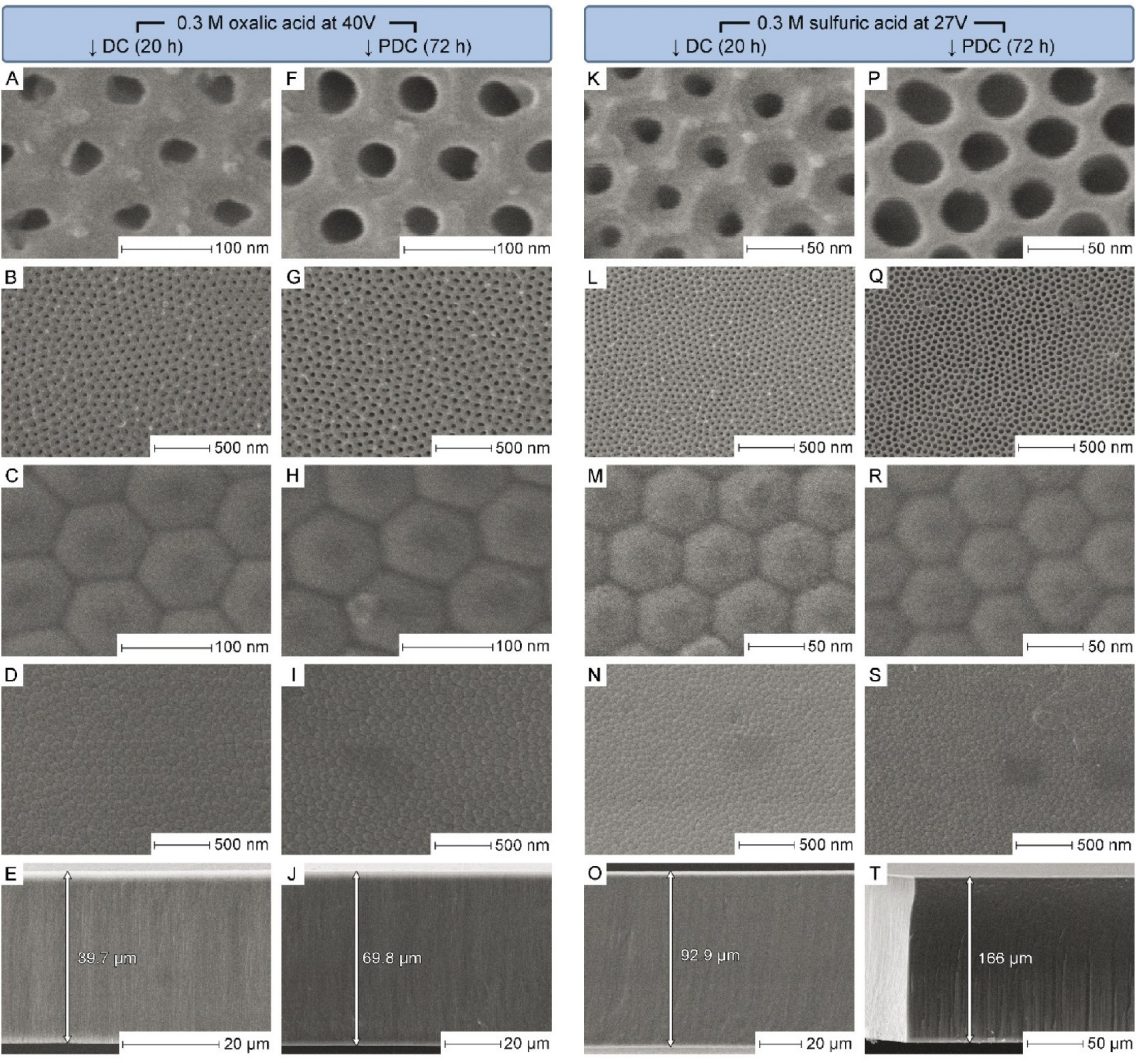
Comparison of SEM micrographs of free-standing PAA layers fabricated in 0.3 M H_2_C_2_O_4_ and 0.3 M H_2_SO_4_ electrolytes using DC and PDC (*f* = 0.1 Hz) anodizing methods. Top surface views (A, F, K, P) show that prolonged 72 h PDC anodizing leads to more pronounced pore widening than 20 h DC anodizing. All samples exhibit semiordered pore arrangements, with PDC-derived films displaying slightly higher pore randomness that may contribute to increased light scattering (B, G, L, Q). Barrier layer views (C, D, H, I, M, N, R, S) confirm clean separation from the metallic substrate achieved via polarity reversal. Cross-sectional images (E, J, O, T) provide film thickness measurements for growth rate estimation.

According to Faraday’s law of electrolysis, PDC-assisted PAA growth should be half as fast as DC, since electrical current passes through the system for only half the anodizing time (assuming equivalent faradaic efficiency in both processes). However, additional considerations introduce uncertainty into this prediction and require experimental verification. First, if the DC-to-PDC transition reduces porosity, then PAA contains a smaller fraction of voids and requires a larger amount of alumina to form films of equivalent thickness. Consequently, PDC-assisted growth rates may differ from DC by a factor greater than 2. Second, only 77% of produced Al^3+^ ions are consumed for DC-generated PAA formation, while 23% are detected in the electrolyte solution.^31^ If this 77/23 ratio increases for low-porosity PDC-derived PAA (i.e., a smaller percentage of Al^3+^ ions is ejected into the solution), then porosity variations may have only negligible influence on PAA growth rates. As shown in Figure 6E,J,O and T, and the data summarized in Table 1, growth rates under DC and PDC regimes differ by factors of approximately 2.05 and 2.01 for oxalic and sulfuric acid electrolytes, respectively. Thus, the systems practically conform to Faraday’s quantitative relationships, and porosity-related factors have only negligible influence.

The faradaic efficiencies of DC and PDC processes can be also compared using the masses *m* of deposited PAA layers corresponding to equivalent electric charges *Q* passed through different types of systems (*m* = *ZQ*, where *Z* is the mass of PAA formed per unit charge; note that gravimetric *m* values may fluctuate due to material loss during anodizing and variable moisture/ionic content in porous adsorptive films, see examples in Figure 7D). However, such a comparison requires several unknown parameters that necessitate estimates and assumptions. First, the fraction of *Q* contributing to anodic alumina growth cannot be reliably estimated for each case due to the poorly quantifiable oxygen evolution reaction (OER) that occurs as the main parasitic side process (e.g., see the theoretical model in which hemispherical pore bottoms are shaped around small bubbles of evolving O_2_ gas, thereby suggesting non-negligible OER activity).^51^ This complicates direct calculation of expected alumina masses. Furthermore, the aforementioned 77/23 ratio between Al^3+^ amounts incorporated into solid and dissolved oxidation products can vary across PAA systems (e.g., due to differing pH conditions at pore bottoms or localized temperature increase due to Joule heating). Faradaic efficiencies in DC and PDC processes can alternatively be compared using masses *m* = *ρV*, where ρ is anodic alumina density and *V* is the net volume of PAA (obtained by subtracting pore volumes from total lamina volume). Since anodic layers consist of X-ray amorphous alumina (ρ = 2.1–3.77 g cm^–3^), boehmite (ρ = 3.0–3.07 g cm^–3^), pseudoboehmite (ρ = 2.4 g cm^–3^), and electrolyte anions (up to 10.56 wt %, see Figure 7) in variable nonstoichiometric proportions, ρ is unknown but can be assumed approximately equal for DC- and PDC-derived PAA.^52^ Under this assumption, the problem reduces to comparing net PAA volumes *V* (*V*_DC_ and *V*_PDC_ for DC- and PDC-derived PAAs, respectively). Using experimentally determined growth rates from Table 1 and porosity values demonstrated in Figure 4D (at *f* = 0.1 Hz) and from Nielsch et al., one can compare net volumes of PAA obtained after passing equivalent amounts of electrical charge through the systems (for this calculation, anodizing durations were set to 1 h for DC and 2 h for PDC regimes).^31^ As shown in Table 2, the net volume ratios *V*_DC_/*V*_PDC_ are less than 1 for both tested electrolytes, with larger deviation for the more aggressive H_2_SO_4_ solution. Combining data from Tables 1 and 2, the faradaic efficiency of metal oxidation in both DC and PDC methods appears practically identical, while the differences in porosities (or in volumes of solid PAA matrices *V*_DC_ and *V*_PDC_) result predominantly from different alumina dissolution dynamics and ejection of different fractions of formed Al^3+^ ions into solution. Assessment through growth rates appears to be an informative complement to the comparison of PAA volume per transferred charge.

**Table 2:** Net Anodic Alumina Volumes *V*_DC_ and *V*_PDC_ Calculated by Subtracting Pore Volumes from Total Volumes of PAA Blocks with a Base Area of 1 μm^2^ (Anodizing Times: 1 h for DC and 2 h for PDC)

PAA type	Porosity *P* (%)	PAA net volume *V* (μm^3^)	Net volume ratio *V*_DC_/*V*_PDC_
0.3 M H_2_C_2_O_4_, DC	8 (ref 31)	1.83	0.99 ± 0.02
0.3 M H_2_C_2_O_4_, PDC (*f* = 0.1 Hz)	4.4 ± 1.6 (this work)	1.85 ± 0.03	
0.3 M H_2_SO_4_, DC	12 (ref 31)	4.09	0.92 ± 0.02
0.3 M H_2_SO_4_, PDC (*f* = 0.1 Hz)	3.8 ± 2.0 (this work)	4.44 ± 0.09	

**Figure 7:**
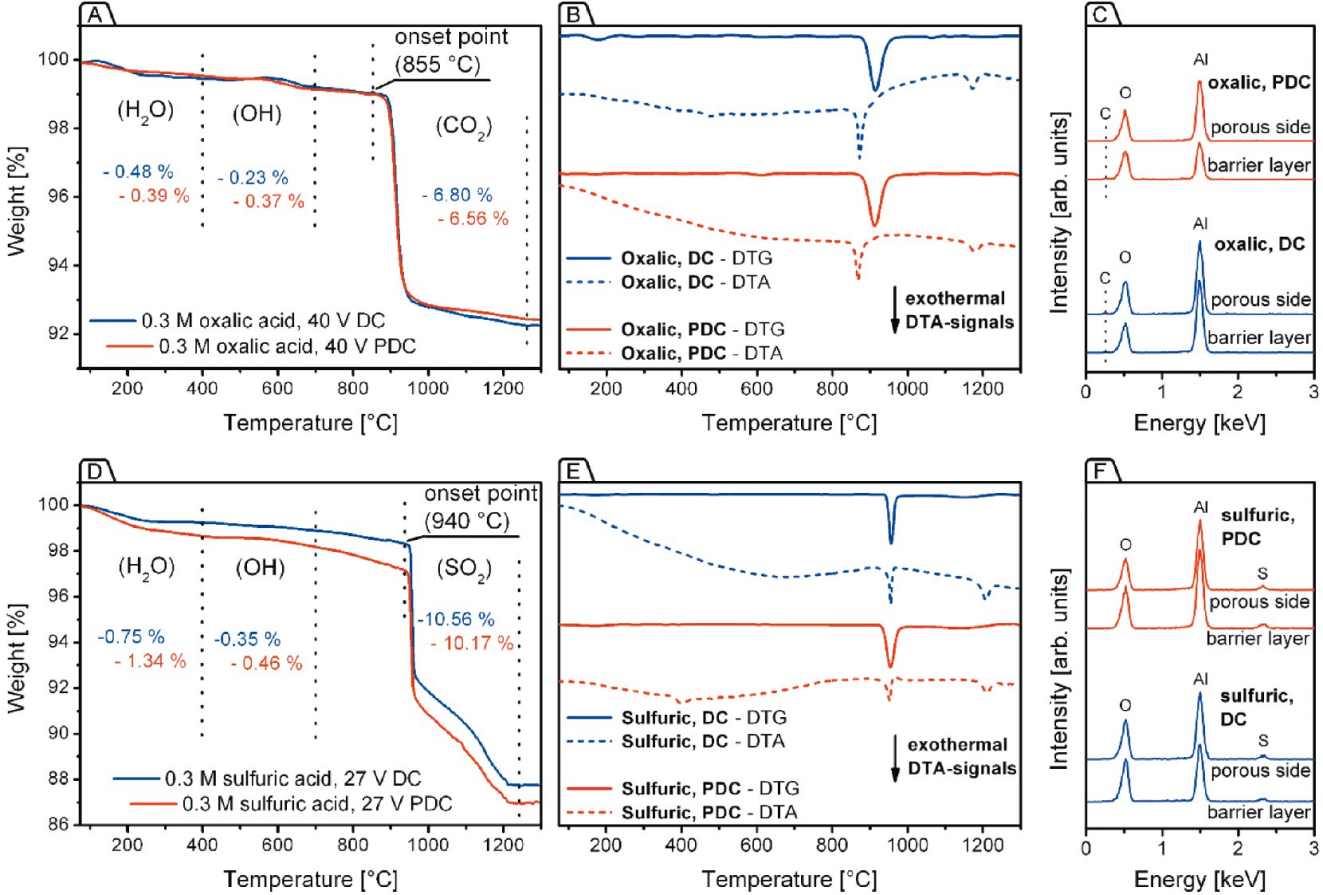
Comparative compositional analysis of DC- and PDC-derived free-standing PAA laminas prepared in 0.3 M H_2_C_2_O_4_ and 0.3 M H_2_SO_4_ electrolytes. For oxalate-containing PAA samples, TGA curves are shown in (A), DTG and DTA curves in (B), and EDX analyses results displaying the elemental compositions on both the porous and basal sides of the unsupported laminas in (C). For sulfate-containing PAA samples, the corresponding TGA, DTG + DTA, and EDX data are presented in (D), (E), and (F), respectively.

To summarize this section, visual observations (Figure 5B) and thickness measurements (Figure 6) show that semiordered PDC-derived “oxalic” films appear more opaque than highly ordered DC-derived “sulfuric” films, despite being about 1.3 times thinner. This indicates that light scattering properties have a complex dependency on morphological factors beyond just the PAA film thickness. The faradaic efficiencies of DC and PDC processes appear essentially equivalent (which manifests itself through the proportionality between *Q* and PAA growth rates), while porosities differ predominantly due to alterations in chemical dissolution dynamics.

### Comparative Compositional Analysis of PDC- and DC-Derived PAA

To examine the nonstoichiometric chemical composition of PDC-derived films and compare it with that of their conventional DC-generated counterparts, comprehensive thermogravimetric (TGA, DTG, and DTA) and energy-dispersive X-ray (EDX) analyses were performed (Figure 7). The data for “oxalic” PAA are shown in Figure 7A–C, while the results for “sulfuric” PAA are presented in Figure 7D–F. The thermal behavior of aluminas obtained from both electrolytes has been extensively studied in previous works.^32,53,54^ Their typical TGA curves (see Figure 7A and D) can be divided into several intervals corresponding to dehydration (from ambient temperature to approximately 400 °C), dehydroxylation (400–700 °C), and acid anion decomposition (with the onset between 820 and 885 °C for oxalate and 920–940 °C for sulfate admixture) steps, often separated by plateau regions of relative thermal stability. These steps can be identified through simultaneous FTIR analysis of volatile decomposition products (such as H_2_O, SO_2_, and CO_2_).^32^ Additionally, transformations, not accompanied by any significant mass losses or composition changes (e.g., phase transitions of alumina), are detectable by DTA. This study does not aim to provide detailed qualitative analysis of the composition, but rather to quantify differences between DC- and PDC-derived laminas. It seeks to determine whether comparable compositions (and related properties) are retained when PDC oxidation is used to modulate PAA morphology.

The TGA curves of DC- and PDC-generated “oxalic” films (Figure 7A) are almost identical across the entire temperature range, with only a minor difference above approximately 1020 °C. This close match indicates that they contain nearly equal amounts of adsorbed water and surface hydroxyl groups. In both cases, the decomposition of oxalate anions begins at ≈885 °C and proceeds in a similar manner. This similarity is well reflected in the DTG curves (Figure 7B), where the “oxalic” samples display nearly identical peaks centered at 914 °C, which corresponds to the highest mass loss rate. Both DTA curves in Figure 7B exhibit broad endothermic processes below 400–450 °C, which correspond to the evaporation of adsorbed water. Three prominent exothermic signals are observed at 475 °C (more pronounced in the DC-generated sample), 870 °C, and 1175–1180 °C. Notably, these exothermic events are not accompanied by significant mass loss in the TGA. This is best illustrated by the mismatch between the most intense DTA and DTG (first-derivative TGA) peaks in the 800–1000 °C range. Therefore, these events should be assigned to restructuring of alumina lattices into more stable polymorph modifications. During calcination of hydrated boehmite (AlO­(OH) – the main component of PAA), γ-alumina typically forms at 350–550 °C and remains stable until approximately 800 °C, after which it transforms sequentially into δ-Al_2_O_3_ (800–900 °C), θ-Al_2_O_3_ (900–1100 °C), and finally α-Al_2_O_3_ (at 1150–1240 °C, depending on the precursor type) phases.^55–58^ Thus, the sharp exothermic peaks observed in the DTA curves correspond to the amorphous → γ, γ → (δ + θ), and θ → α alumina phase transitions. The decomposition of oxalate anions in the absence of oxygen is known to be an endothermic process, which explains the curvature of the DTA plots starting from approximately 900 °C. The residual mass of the “oxalic” films (Figure 7A) is 92.3–92.4 wt %, with 0.7–0.8 wt % loss due to removal of adsorbed and structural water and surface hydroxyl groups, and 6.6–6.8 wt % due to oxalate ions decarboxylation and CO_2_ release. Based on these TGA results, the estimated carbon content (excluding the oxygen from CO_2_) is below 2 wt % for both DC- and PDC-generated samples. However, EDX analysis (Figure 7C) suggested significantly higher atomic percentages of carbon. For DC-generated PAA, the C content was 6 ± 1 wt % on both the porous and basal (nonporous) sides. For PDC-generated PAA, it was 5 ± 2 and 10 ± 1 wt %, respectively. This discrepancy between thermogravimetric and spectroscopic methods likely stems from EDX instrumental limitations. First, EDX cannot detect elements lighter than boron, making hydrogen from H_2_O and OH-groups invisible and increasing the percentages of detectable elements above their actual values. Moreover, C is close to the detection limit, causing substantial errors in standardless quantification (exceeding the typical 1 wt % for heavier elements). It should also be taken into account that EDX is a surface-sensitive method, and therefore it may reflect an actual increase in C-content due to surface-adsorbed oxalate ions or adventitious carbon, although any contribution from the latter is expected to be negligible (significantly less than in XPS). Therefore, TGA results should be considered more reliable for carbon content determination.

As shown in Figure 7D, samples prepared in 0.3 M H_2_SO_4_ exhibit noticeably different quantitative ratios of their main components. In PDC-derived “sulfuric” films, the H_2_O and OH-groups contents are 79% and 31% higher, respectively, than in DC-derived PAA, while the sulfate impurity content is almost 4% lower. These findings are consistent with the SEM comparison in Figure 6K and P: the larger *d*_p_ values observed in PDC-derived samples result in greater pore volume and surface area, leading to higher water and surface hydroxyl groups content. The lower sulfate percentage can be explained by selective dissolution of electrolyte-contaminated zones during anodization. Every PAA cell consists of two sections: an electrolyte-rich area near the pore interior and relatively pure alumina where neighboring cells are adjacent.^59^ Prolonged anodizing leads to predominant dissolution of the contaminated regions due to their direct exposure to the electrolyte, while pure alumina remains protected. Consequently, the final PAA contains a smaller share of sulfate-containing material, reflected in the correspondingly lower mass loss in TGA. The three sharp exothermic peaks in the DTA curves (Figure 7E) at 400, 952, and 1210 °C are shifted toward higher temperatures compared to “oxalic” aluminas. This shift places the γ → θ transition peak very close to the sulfate decomposition event in DTG, potentially complicating interpretation. However, this sharp DTA signal is superimposed with a broad endothermic event spanning from ≈650 to ≈1200 °C that actually corresponds to SO_4_^2–^ decomposition. TGA results indicate that the sulfur content (excluding the oxygen from SO_2_) in both samples analyzed in Figure 7D is 5.1–5.3 wt %. In contrast, EDX analysis (Figure 7F) detected 3 ± 1 wt % of sulfur on both sides of DC- and PDC-generated laminas. The discrepancy between thermogravimetry and EDX values is notably smaller than observed for carbon impurities. However, this difference cannot be attributed solely to instrumental limitations. The variation in S content between bulk material and surface (the EDX analysis depth is ≈2 μm) may result from the efficient removal of H_2_SO_4_ solution from PAA surfaces during postsynthesis washing with deionized water, while residual liquid trapped within nanochannels remains and contributes to the total sulfur content measured after drying.

## Conclusions

The results of this experimental study demonstrate that square-wave PDC anodizing is a straightforward technique with the potential to set new standards for flexibility and precision in the morphological tuning of PAA architectures. This method enables two-level structural control, allowing largely decoupled adjustment of pore diameter *d*_p_ and the degree of hexagonal ordering, while maintaining nearly constant interpore distances and pore densities (*D*_int_ variations within 9–25%). Upon transition from DC to PDC anodizing, the PAA retains its characteristic honeycomb-like structure without a noticeable increase in nanochannel defects, while preserving its nonstoichiometric chemical composition. The PDC-assisted growth rate is governed by Faraday’s law and is approximately half that observed under conventional potentiostatic conditions. These factors make the new morphological tailoring approach highly predictable and reliable, offering potential universality across various electrolytes (the basic principles have been demonstrated in 10 wt % H_2_SO_4_ at 20 V in ref 26, in 0.3 M H_2_SO_4_ at 27 V and in 0.3 M H_2_C_2_O_4_ at 40 V in this work, with further evidence to be gathered using other electrolytes not previously tested under PDC conditions) and significant flexibility in PAA geometry manipulation.

When anodizing duration is kept within optimized limits, the resulting PDC-generated PAA is not subject to critical pore widening due to alumina dissolution (where “critical” refers to dissolution that negates the PDC-induced pore contraction). After 3 h of anodizing, the 2.9 μm thick “oxalic” and 6.9 μm thick “sulfuric” laminas exhibited up to 1.8-fold and 2.4-fold reductions in *d*_p_, respectively. Notably, the surface porosity of PAA obtained from 0.3 M H_2_SO_4_ decreased by a factor of 5 (at *f* = 0.5 Hz) compared to DC-generated counterparts, which is a remarkable outcome considering that no complex aluminum surface pretreatment was required. A more detailed study aimed at quantifying PAA dissolution rates may involve practical difficulties and require individual testing of each set of anodizing conditions. As summarized in a recent review by Martín-González, pore dissolution kinetics is nonlinear and may vary with chemical environment changes over time due to electrolyte “aging” (accumulation of Al^3+^ ions in solution).^7^ Additionally, dissolution rates may depend on varying “chemical reactivities” of PAA samples obtained at different specific voltages (this difference is most clearly observed when comparing “mild anodized” versus “hard anodized” layers).^7^

Both investigated types of PDC-derived PAA laminas exhibit analogous self-ordering behavior: by adjusting the pulse frequency *f* while keeping other anodization parameters constant, similar nonlinear evolution dynamics are observed, characterized by alternating stages of nucleation and growth (merging) of hexagonally ordered nanopore domains. While further studies are needed to statistically validate these self-ordering patterns, it is already evident that the “frequency-based” approach offers new opportunities for optical tunability. The degree of pore ordering correlates with the macroscopically observed transparency of free-standing PAA laminas, which is promising for the development of anisotropic light-scattering metamaterials.

Future research should focus on exploring additional anodization parameters (e.g., duration, electrolyte temperature) in combination with fixed pulse frequencies *f* to statistically substantiate their influence and define the boundaries of this new pore array engineering approach. Additionally, linking pore structure tunability to specific applications in photonics (2D crystals, disordered lattices with a deliberately controlled degree of irregularity), plasmonics, or SERS will be an important direction for continued investigation. For example, given the large decrease in porosity upon the DC-to-PDC transition with only moderate changes in the periodicity of nanopore arrangement, it would be beneficial to explore mixed DC and PDC anodizing regimes for fabricating multilayer PAA structures with sequential *P* and *n*_eff_ contrast, which are of current interest for distributed Bragg reflectors.^60^ A comparative study of PAA systems where current oscillations arise either from imposed voltage pulses or spontaneously in a DC process can also be of interest (a brief discussion of spontaneous oscillations can be found elsewhere).^61^ Since electrodes and electrolyte solutions have different types of electrical conductivity (electronic and ionic, respectively), the resulting current density depends on charge carrier conversion rates at electrode/electrolyte interfaces. Under fixed applied potential and bath temperature, these conversion rates vary primarily due to reactant concentration fluctuations near the electrode surface (according to the law of mass action). The nature of such concentration fluctuations has received explanation through competition between Coulomb forces on ions and their oppositely directed diffusion.^62^ More generally, electrochemical reaction rates are controlled by activation overpotentials and concentration and resistance polarization effects. In other words, current density oscillations can result not only from ionic concentration fluctuations but also from fluctuations in the ohmic resistance of the dynamically evolving anodic alumina layer. It has been recently demonstrated that such spontaneous oscillations (including the damped oscillations described earlier in ref 62) can emerge during DC anodization of diluted Al–Cu alloys at relatively low applied voltages (Δ*U* > 30 V), creating “horizontal” interconnections between the main “vertical” pores.^63^ This exemplifies the complex multifaceted nature of PAA systems, the interplay between controlled parameters and spontaneously occurring phenomena, and the diversity of possible formation mechanisms in a dynamic ionic environment. The PDC-based PAA engineering approach demonstrated in this work extends the scientific knowledge base for further systematic exploration of such nontrivial process-morphology relationships.

## Supplementary Material



## Notes

Quantitative analysis of ordered pore domain surface areas from SEM images of PDC-anodized PAA layers at all tested frequencies, including detailed image analysis methodology; and statistical analysis of PAA morphological parameters presented on a logarithmic *f* scale for improved visualization of low-frequency data (PDF)
