# Supplementary material for: Expanding the Porous Alumina Engineering Toolbox for Nanooptics: Multiscale Morphological Tuning via Square-Wave Pulse Anodizing
Source: ACS Omega. 2026 Apr 14;11(16):24808–21. doi: 10.1021/acsomega.6c02015 (PMC13130112; doi:10.1021/acsomega.6c02015)
Supplement: Supplementary file 1 [file ao6c02015_si_001.pdf]

Supporting Information

Expanding the Porous Alumina Engineering Toolbox for Nanooptics: Multiscale Morphological Tuning via Square-Wave Pulse Anodizing

Mikhail Pashchanka

Department of Chemical Engineering, Ariel University, Ariel 40700, Israel; mikhailpa@ariel.ac.il.

Experimental Conditions:

Pulse anodizing of Al sheets was performed in 0.3 M oxalic acid ($H_2C_2O_4$) and 0.3 M sulfuric acid (H_2SO_4) electrolyte solutions at maximum output voltages of 40 V and 27 V, respectively. A preliminary oxidation step (4 h) was followed by removal of PAA using a wet etching mixture and a main step (3 h). For full details, refer to the main manuscript.

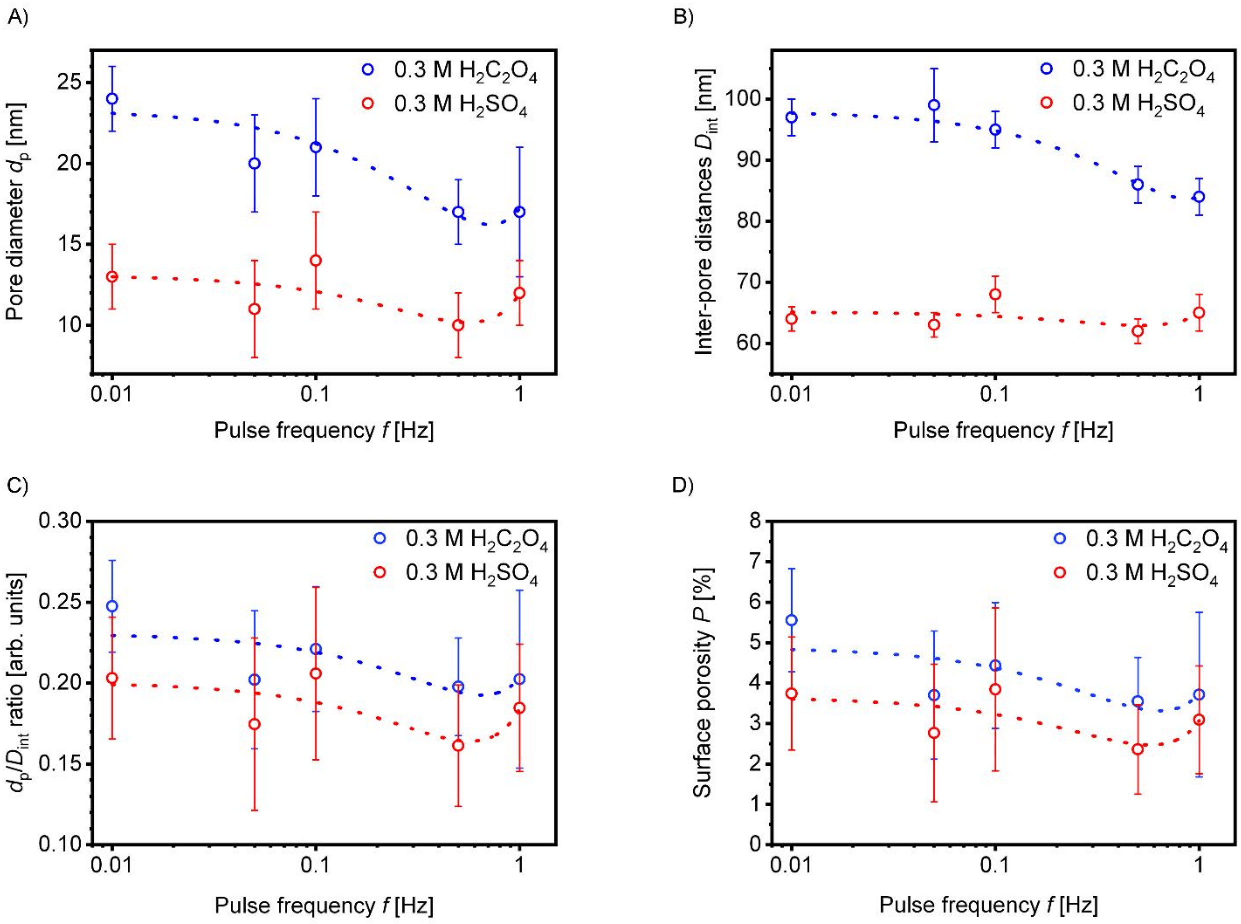

Figure S1. Statistical analysis of pore diameter d_p(A), interpore spacing D_{int} (B), calculated d_p/D_{int} ratios (C), and surface porosity P (D) as functions of pulse frequency. Pulse frequencies f are shown on a logarithmic scale to improve visualization of statistical variance and deviations from the fitted curves in the low-frequency range.

Quantitative Analysis of Hexagonally Ordered Pore Domain Surface Areas in PDC-Anodized PAA Layers

Well-ordered pore domain surface areas were quantified in SEM images of porous anodic alumina (PAA) layers fabricated via pulsed direct current (PDC) anodizing (rectangular waveform, f = 1, 0.5, 0.1, 0.05, and 0.01 Hz).

Image Analysis:

All calculations were performed using the SketchAndCalc irregular area calculator – a precise tool that meets technical and scientific standards (https://www.sketchandcalc.com/).
Every micrograph (80 000× magnification) had a total visible surface area of approximately **3 974 000 nm²** (scale bar insets were excluded from calculation). Ordered domain fractions (%) were calculated relative to this total area. Analysis of PAA samples obtained under each set of experimental conditions was performed using a single SEM image. Therefore, no statistical data treatment was applied. To conclusively establish the evolution patterns of self-ordering, further dedicated studies with repeated experiments are necessary.

Analysis of PAA Layers Obtained by Anodizing Aluminum in 0.3 M Oxalic Acid ($H_2C_2O_4$) Electrolyte Solution (PDC, ΔU = 40 V)

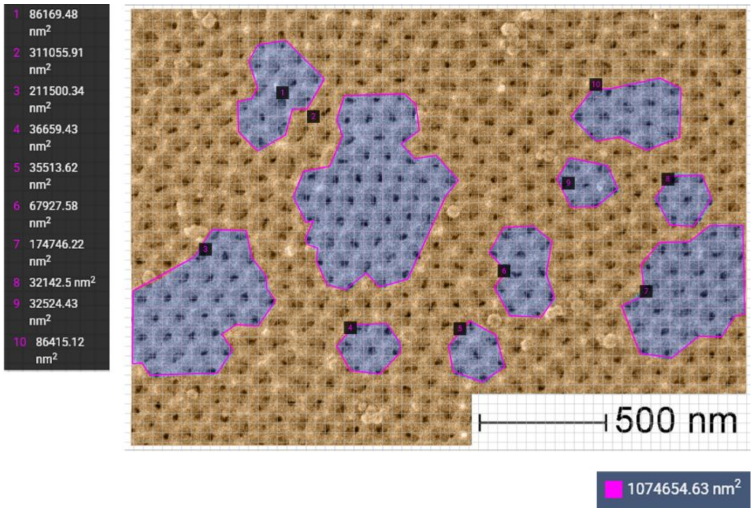

Figure S2. 0.3 M $H_2C_2O_4$, Pulse frequency f = 1 Hz

Sum of ordered domain surface areas:
1 074 655 nm²

Surface area fraction of ordered domains:
27.0 %

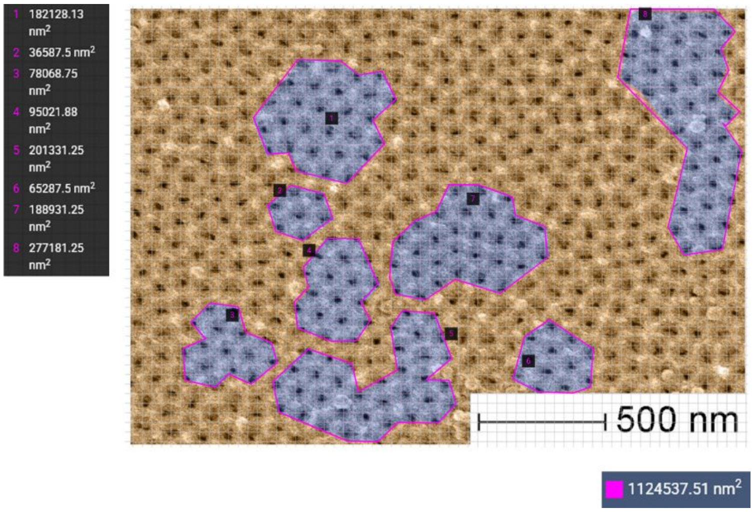

Figure S3. 0.3 M $H_2C_2O_4$, Pulse frequency f = 0.5 Hz

Sum of ordered domain surface areas:
1 124 538 nm²

Surface area fraction of ordered domains:
28.3 %

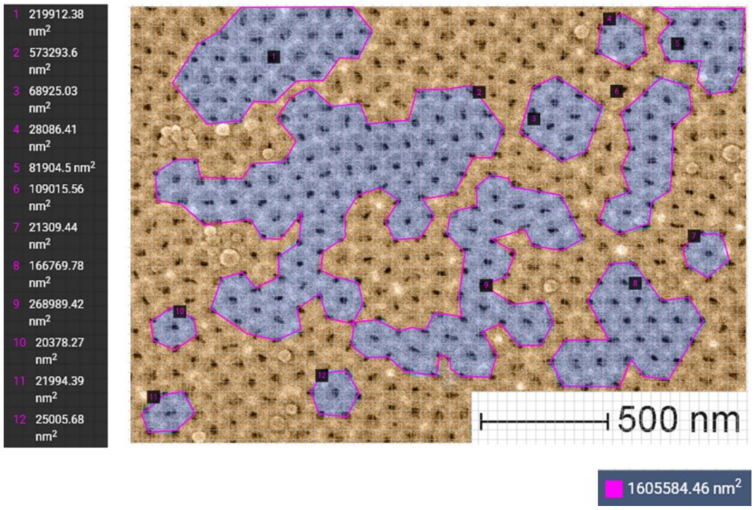

Figure S4. O.3 M $H_2C_2O_4$, Pulse frequency *f* = 0.1 Hz

Sum of ordered domain surface areas: 1 605 584 nm^2

Surface area fraction of ordered domains: **40.4 %**

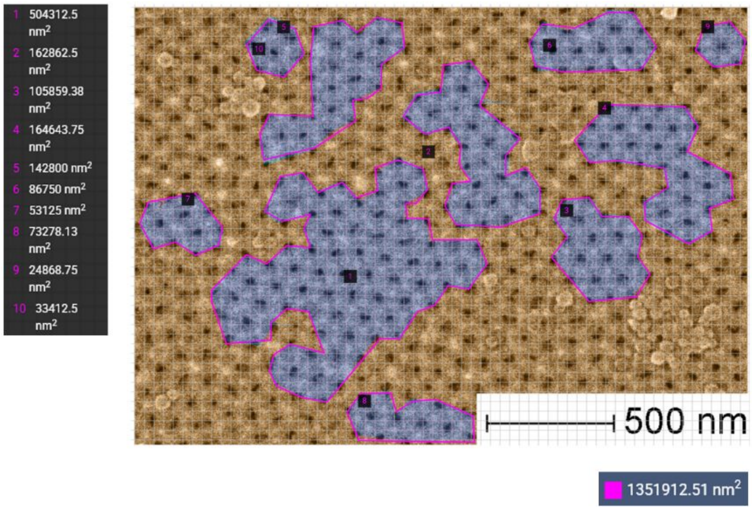

Figure S5. O.3 M $H_2C_2O_4$, Pulse frequency *f* = 0.05 Hz

Sum of ordered domain surface areas: 1 351 913 nm^2

Surface area fraction of ordered domains: **34.0 %**

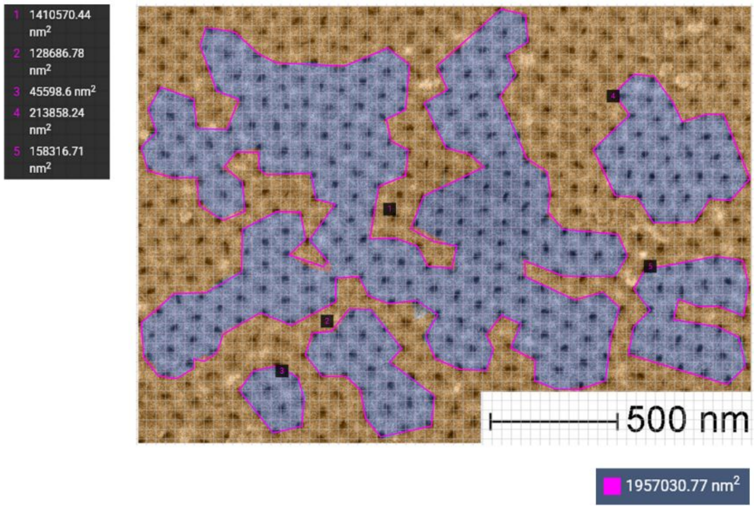

Figure S6. O.3 M $H_2C_2O_4$, Pulse frequency *f* = 0.01 Hz

Sum of ordered domain surface areas: 1 957 031 nm^2

Surface area fraction of ordered domains: **49.2 %**

Analysis of PAA layers Obtained by Anodizing Aluminum in 0.3 M Sulfuric Acid (H_2SO_4) Electrolyte Solution (PDC, ΔU = 27 V)

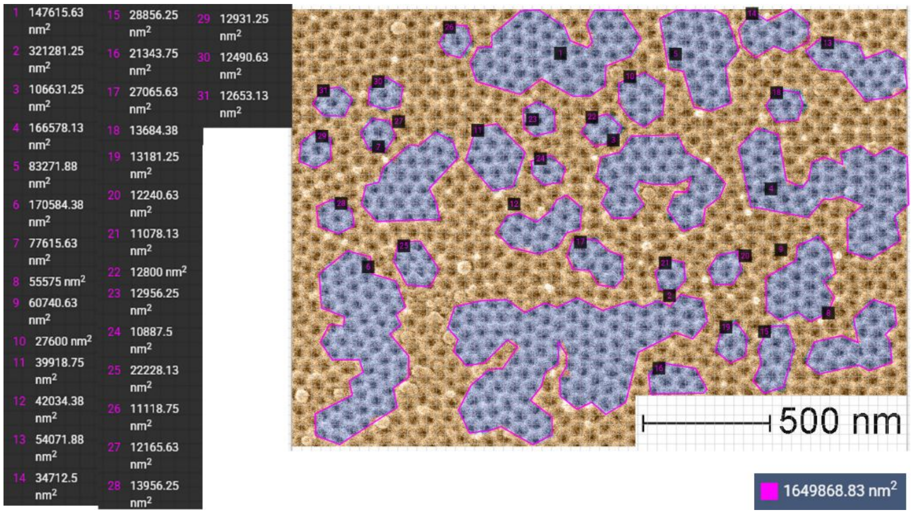

Figure S7. O.3 M H_2SO_4, Pulse frequency f = 1 Hz

Sum of ordered domain surface areas: 1 649 869 nm^2

Surface area fraction of ordered domains: **<u>41.5 %</u>**

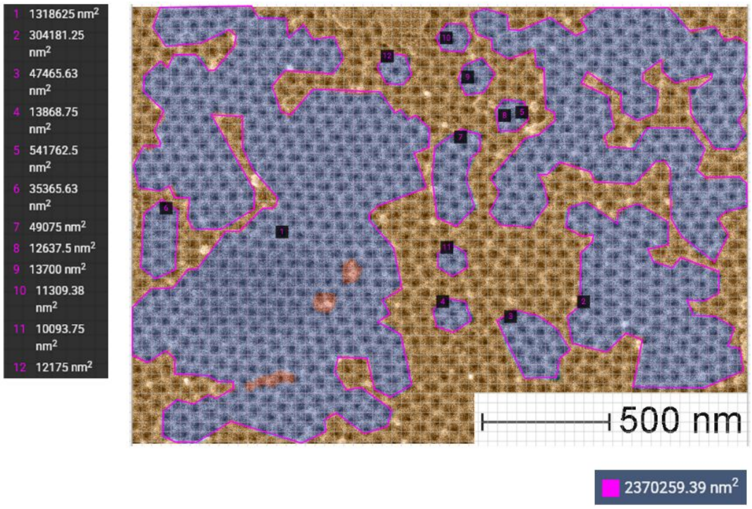

Figure S8. O.3 M H_2SO_4, Pulse frequency f = 0.5 Hz

Sum of ordered domain surface areas: 2 370 259 nm^2

Surface area fraction of ordered domains: **<u>59.6 %</u>**

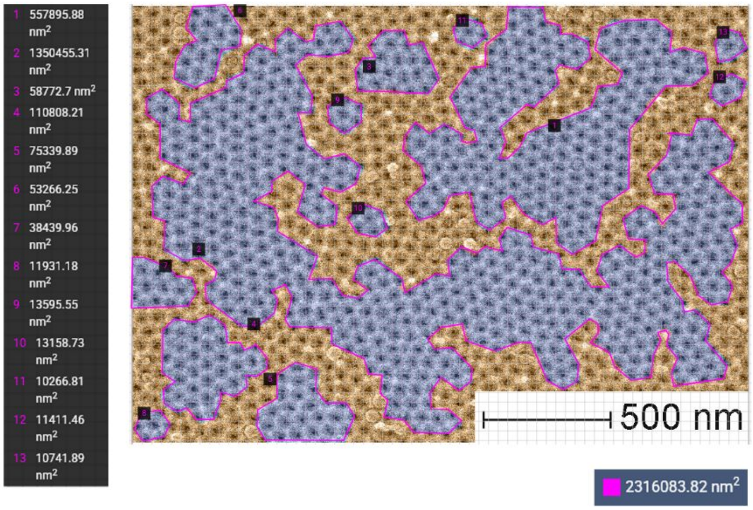

Figure S9. O.3 M H_2SO_4, Pulse frequency f = 0.1 Hz

Sum of ordered domain surface areas: 2 316 084 nm^2

Surface area fraction of ordered domains: **<u>58.3 %</u>**

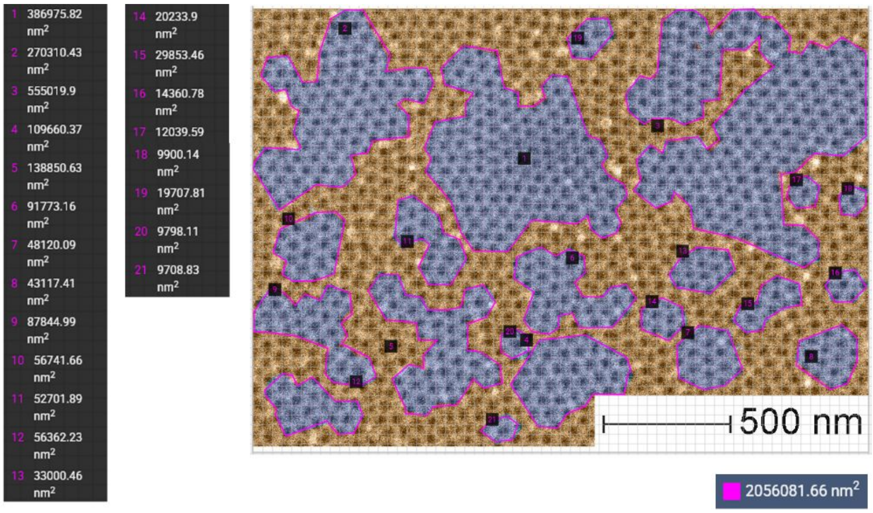

Figure S10. O.3 M H_2SO_4, Pulse frequency f = 0.05 Hz

Sum of ordered domain surface areas:
2 056 082 nm^2

Surface area fraction of ordered domains:
51.7 %

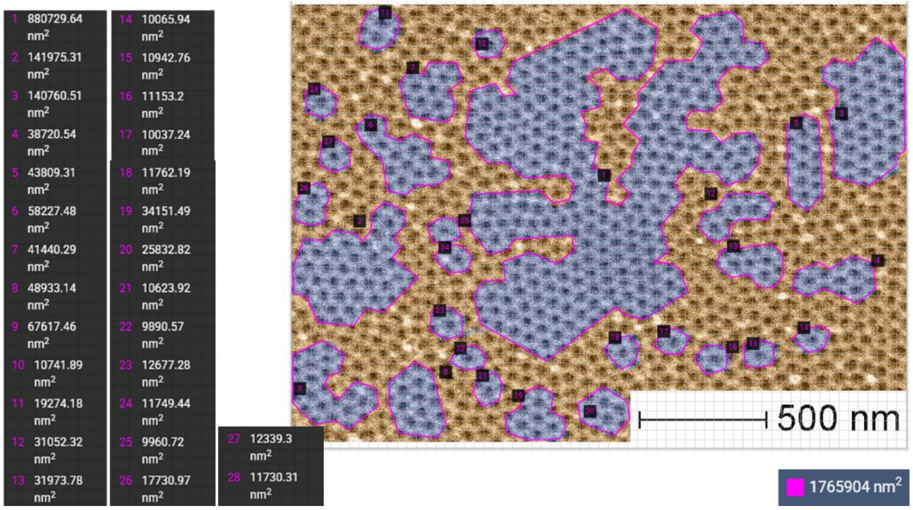

Figure S11. O.3 M H_2SO_4, Pulse frequency f = 0.01 Hz

Sum of ordered domain surface areas:
1 765 904 nm^2

Surface area fraction of ordered domains:
44.4 %
